# *Hydra myc2*, a unique pre-bilaterian member of the *myc* gene family, is activated in cell proliferation and gametogenesis

**DOI:** 10.1242/bio.20147005

**Published:** 2014-04-25

**Authors:** Markus Hartl, Stella Glasauer, Taras Valovka, Kathrin Breuker, Bert Hobmayer, Klaus Bister

**Affiliations:** 1 Institute of Biochemistry, University of Innsbruck, A-6020 Innsbruck, Austria; 2 Center for Molecular Biosciences Innsbruck (CMBI), University of Innsbruck, A-6020 Innsbruck, Austria; 3 Institute of Zoology, University of Innsbruck, A-6020 Innsbruck, Austria; 4 Institute of Organic Chemistry, University of Innsbruck, A-6020 Innsbruck, Austria; * Present address: Institute of Molecular Life Sciences, University of Zurich, CH-8057 Zurich, Switzerland.

**Keywords:** Cnidarian, Oncogenic transcription factor, Gene regulation, Development, Evolution

## Abstract

The *myc* protooncogene encodes the Myc transcription factor which is the essential part of the Myc–Max network controlling fundamental cellular processes. Deregulation of *myc* leads to tumorigenesis and is a hallmark of many human cancers. We have recently identified homologs of *myc* (*myc1*, *myc2*) and *max* in the early diploblastic cnidarian *Hydra* and have characterized *myc1* in detail. Here we show that *myc2* is transcriptionally activated in the interstitial stem cell system. Furthermore, in contrast to *myc1*, *myc2* expression is also detectable in proliferating epithelial stem cells throughout the gastric region. *myc2* but not *myc1* is activated in cycling precursor cells during early oogenesis and spermatogenesis, suggesting that the *Hydra* Myc2 protein has a possible non-redundant function in cell cycle progression. The Myc2 protein displays the principal design and properties of vertebrate Myc proteins. In complex with Max, Myc2 binds to DNA with similar affinity as Myc1–Max heterodimers. Immunoprecipitation of *Hydra* chromatin revealed that both Myc1 and Myc2 bind to the enhancer region of *CAD*, a classical Myc target gene in mammals. Luciferase reporter gene assays showed that Myc1 but not Myc2 transcriptionally activates the *CAD* promoter. Myc2 has oncogenic potential when tested in primary avian fibroblasts but to a lower degree as compared to Myc1. The identification of an additional *myc* gene in Cnidaria, a phylum that diverged prior to bilaterians, with characteristic expression patterns in tissue homeostasis and developmental processes suggests that principle functions of *myc* genes have arisen very early in metazoan evolution.

## INTRODUCTION

The *myc* gene was originally identified as oncogenic determinant (v-*myc*) in the genome of avian acute leukemia virus MC29 (Duesberg et al., 1977; Bister and Jansen, 1986; Vogt, 2012; Conacci-Sorrell et al., 2014). The v-*myc* oncogene is derived from the cellular c-*myc* protooncogene encoding a master regulator (Myc) of fundamental cellular processes like growth, proliferation, differentiation, metabolism, and apoptosis (Eilers and Eisenman, 2008; Dang, 2012; Conacci-Sorrell et al., 2014). Other members of the vertebrate *myc* gene family are B-*myc*, L-*myc*, N-*myc*, and s-*myc* (Eisenman, 2001; Eilers and Eisenman, 2008; Dang, 2012). Myc represents a transcription regulator controlling the expression of numerous target genes which are involved in multiple cellular processes such as ribosomal biogenesis, protein biosynthesis, cell cycle control, or energy metabolism (Eisenman, 2001; Eilers and Eisenman, 2008; Dang, 2012; Conacci-Sorrell et al., 2014). Deregulation of human *myc* genes caused by transcriptional activation, gene amplification, or chromosomal translocation results in elevated Myc protein levels which contribute to tumorigenesis (Dalla-Favera et al., 1982; Nesbit et al., 1999; Dang, 2012) presumably by aberrant Myc target gene regulation (Eisenman, 2001; Eilers and Eisenman, 2008; Dang, 2012). Therefore, deregulated *myc* can be regarded as a hallmark of many human cancers (Hanahan and Weinberg, 2011). Myc is a bHLH-Zip (basic region, helix–loop–helix, leucine zipper) protein which dimerizes with bHLH-Zip partner proteins like Max upon specific binding to DNA sequence elements termed E-boxes (Eilers and Eisenman, 2008). Besides extensive characterization of Myc and Max protein family members in vertebrates, orthologs have also been identified in invertebrates like the fruit fly *Drosophila* (Gallant, 2006). Like in vertebrates, *Drosophila* Myc controls the expression of many genes that are involved in ribosome biogenesis, protein synthesis, and metabolism (Bellosta and Gallant, 2010), suggesting that the principal functions of the Myc transcription factor network have been conserved through evolution (Conacci-Sorrell et al., 2014). Recent investigations using even simpler invertebrate systems led to the identification and isolation of ancestral *myc* and *max* genes from diploblastic cnidarians like *Hydra* (Hartl et al., 2010; Hobmayer et al., 2012) or *Hydractinia* (Millane et al., 2011), and from the choanoflagellate *Monosiga brevicollis* (Young et al., 2011), which represent the most early diverged species employed so far for the structural, functional, and evolutionary analysis of Myc. The *Hydra myc1* gene was found to be specifically activated in all interstitial stem cells and nematoblast nests, which represent the rapidly proliferating cell types of the interstitial stem cell system, and in proliferating gland cells. Inhibition of *myc1* impairs the balance between stem cell self-renewal and differentiation (Ambrosone et al., 2012). In terminally differentiated nerve cells, nematocytes, or epithelial cells, *myc1* expression is not detectable. By analyzing the *Hydra* genome, we have also obtained evidence for the presence of a second functional *Hydra myc* gene termed *myc2* being located immediately adjacent to the *Hydra CAD* gene in head-to-head orientation (Hartl et al., 2010). *CAD* encodes the multifunctional enzyme carbamoylphosphate synthetase/aspartate transcarbamoylase/dihydroorotase which is required for pyrimidine biosynthesis and is known as a bona fide transcriptional target of mammalian Myc proteins (Miltenberger et al., 1995; Boyd and Farnham, 1997; Dang, 1999). Here, we describe the structural and functional analysis of the *Hydra myc2* gene and its protein product, and compare its expression pattern during *Hydra* development with that of *myc1*. We show that like *myc1*, *myc2* is strongly expressed in proliferating interstitial cells but in addition, *myc2* is also transcriptionally activated in epithelial cells and during gametogenesis. Like Myc1, the Myc2 protein displays the principal structure, and the typical biochemical and oncogenic functions of its vertebrate derivatives suggesting that general functions of the Myc master regulator have evolved very early.

## MATERIALS AND METHODS

### Animals

*Hydra vulgaris* strains Basel, AEP, PA-2 and *Hydra magnipapillata* strains 105 and A10 were used in this study in compliance with animal welfare laws and policies. Mass cultures were kept at 18°C and fed five times per week with freshly hatched *Artemia nauplii* as described (Hobmayer et al., 1997). Experimental animals were collected 24 h after the last feeding. To induce the formation of eggs in the AEP strain and of testes in the PA-2 strain, a starvation stimulus was used as described (Hobmayer et al., 2001). To obtain interstitial cell free polyps, *Hydra* of the mutant A10 strain were treated as follows: approximately 150 animals were collected, fed and washed two hours after feeding. Shortly after that, a heat-shock was started by placing the animals into an incubator adjusted to 26°C, where they stayed for 48 hours. During and after the heat-shock, daily feeding was continued. Eight days after beginning of the heat-shock, the animals were collected for RNA isolation. At that time point, total loss of proliferating interstitial cells (Marcum et al., 1980) was determined in maceration preparations (David, 1973).

### DNA cloning and nucleic acid analysis

Molecular cloning, DNA sequencing, and Northern analysis have been described (Hartl et al., 2009; Hartl et al., 2010). RNA isolation from whole *Hydra* animals, poly(A)^+^-RNA selection, whole mount *in situ* hybridization with digoxigenin-labeled RNA probes, and double *in situ* hybridization using digoxigenin- or fluorescein-labeled RNA probes were done as described (Philipp et al., 2005; Hartl et al., 2010). Bioinformatic search of the *Hydra* database using mammalian CAD protein sequences as templates lead to the identification of sequence contigs (NW_002159242, NW_002118084, NW_002173794, NW_002105431, NW_002159544) related to vertebrate *CAD* genes. One contig (accession no. NW_002159242) contains the presumed translation start site of a putative *Hydra* CAD protein. With this information, the full-length coding region of *CAD* was cloned by using poly(A)^+^-selected RNAs isolated from whole *Hydra magnipapillata* animals as a template for cDNA synthesis, followed by polymerase chain reaction (PCR) and the primer pairs 5′-ATGGCAAAATTAGTGTTGGAAAATGGAAG-3′/5′-TTAATATCGACCCGTTAACATACATAGTAAAG-3′. The PCR product was inserted into the *Sma*I site of the pUC19 vector and sequenced. To determine the *Hydra myc2* or *CAD* transcription start sites, 5′-rapid amplification of cDNA ends (RACE) was performed as described (Reiter et al., 2007) using the primers 5′-GTGTACACCAATTTGAACCAGTCATATCGA-3′ and 5′-AATTTATCCACAGCTATTATGTACACAATT-3′ (*myc2*), or 5′-CAAATCTCCAACAACTAAACCACTTGTCC-3′ and 5′-CCCTTTAAACTCACTTCCATTTTCCAACAC-3′ (*CAD*) for first strand cDNA synthesis and subsequent PCR, respectively. Cloning of the *Hydra myc1*, *myc2*, and *max* coding regions by cDNA synthesis and PCR has been described (Hartl et al., 2010).

### Cells and retroviruses

Cultivation of quail embryo fibroblasts (QEF) and of the methylcholanthrene-transformed cell line QT6, calcium phosphate-mediated DNA transfection, and cell transformation assays were performed as described (Reiter et al., 2007; Hartl et al., 2009; Hartl et al., 2010). The constructs pBS-hymyc1, pBS-hy1/v-myc, pBS-v/hy1-myc, pBS-v-myc, pRCAS-hy1/v-myc, pRCAS-v/hy1-myc, and pRCAS-v-myc have been described (Hartl et al., 2010). To construct the plasmids pA-HM2-VM and pA-VM-HM2 encoding hybrid proteins of *Hydra* Myc2 (HM2) and v-Myc (VM), the corresponding *myc2* and v-*myc*-specific segments containing overlapping sequences were amplified in four different PCRs using the adaptor plasmids pA-hymyc2 and pA-v-myc (Hartl et al., 2010) as templates and the primer pairs 5′-GGCCCTTTCGTCTTCAAG-3′/5′-GTGCGTTCGCCTCTTGTCAATTAACGCTGATTTACG-3′ (HM2a), 5′-CGTAAATCAGCGTTAATTGACAAGAGGCGAACGCAC-3′/5′-ATGGAAAAACGCCAGCAAC-3′ (VMb), 5′-GGCCCTTTCGTCTTCAAG-3′/5′-AGTAGGATCTAAATCATCGTTCTCCTCTGAGTCTAA-3′ (VMa), 5′-TTAGACTCAGAGGAGAACGATGATTTAGATCCTACT-3′/5′-ATGGAAAAACGCCAGCAAC-3′ (HM2b). In two subsequent PCRs, diluted pools from pairs of the first PCRs were employed as templates (HM1a + VMb and VMa + HM2b) using the primer pairs 5′-GGCCCTTTCGTCTTCAAG-3′/5′-ATGGAAAAACGCCAGCAAC-3′. The resulting PCR products were digested with *Xba*I (HM2-VM) or *Sal*I (VM-HM2), and inserted into pA-CLA12NCO or pA-v-*myc* which had been opened with *Xba*I or *Sal*I, respectively. The inserts of pA-hymyc2, pA-HM2-VM, and pA-VM-HM2 were released with *Cla*I and inserted into the retroviral RCAS-BP vector, or into the Bluescript pBS SKII+ vector for *in vitro* translation as described (Hartl et al., 2010).

### Recombinant protein expression, purification, and mass spectrometry

Expression and purification of recombinant *Hydra* Max and Myc1 p16 proteins has been described (Hartl et al., 2010). To generate the prokaryotic expression vectors pET11d-hymyc2 and pET11d-p15hymyc2, *Hydra myc2*-specific coding regions were amplified from cDNAs. The PCR segments encompassing the full-length *Hydra myc2* coding sequence (hymyc2), or the region encoding amino acids 223–327 (p15hymyc2) were inserted into the pET11d vector (Novagen, Darmstadt, Germany) as described (Hartl et al., 2010).

For the expression of full-length Hydra Myc2 protein (327 amino acids; *M*_r_ = 36,961; pI = 5.99), the plasmid pET11d-hymyc2 was transformed into *Escherichia coli* strain BL21 (DE3) CodonPlus-RIL (Stratagene, Santa Clara, CA). An overnight culture in LB broth supplemented with 100 µg/ml ampicillin was diluted 1:100 to yield a 400-ml culture. Bacteria were grown at 37°C to an optical density of 0.6 at 600 nm, recombinant protein synthesis was induced by the addition of isopropyl-β-D-thiogalactoside (IPTG) to a final concentration of 1 mM, and incubation was continued for 4 h. The bacterial pellet was resuspended in 20 ml ice-cold buffer B (20 mM Tris-HCl pH 7.5, 1 M NaCl, 1 mM DTT, 1 mM PMSF) and lysed at 1,300 psi using a French Press. To lower the viscosity, the cell extract was supplemented with 1.7 µg/ml *DNase* I and 0.3 mM MgCl_2_, and incubated for 30 min on ice. The sample was then centrifuged at 18,000 × g for 20 min at 4°C. The pellet was re-suspended in buffer A (20 mM Tris-HCl, pH 7.5, 80 mM NaCl, 1 mM EDTA, 1 mM DTT), supplemented with 0.05% (w/v) sodium deoxycholate, and sonicated for 15 sec. The inclusion bodies containing the recombinant protein were pelleted at 18,000 × g for 30 min at 4°C. Inclusion bodies were washed with buffer A supplemented with 0.05% (w/v) sodium deoxycholate and 1% (v/v) NP-40, and then with buffer A. Inclusion bodies were suspended in buffer A supplemented with 7 M urea and separated on a Mono Q 5/50 GL anion exchange column (GE Healthcare, Little Chalfont, UK) in the presence of 7 M urea as described (Hartl et al., 2010) using an ÄKTA Purifier (GE Healthcare). Proteins were eluted using a linear gradient from 0 to 0.5 M NaCl at a flow rate of 0.5 ml/ml. High salt elution fractions containing the purified recombinant *Hydra* Myc2 protein were pooled and dialyzed against 1 l of PBS (140 mM NaCl, 2.7 mM KCl, 10 mM Na_2_HPO_4_, 1.8 mM KH_2_PO_4_) containing 1 mM DTT for 12 h at 4°C, and then stored in liquid nitrogen. The final yield of purified and soluble *Hydra* Myc2 protein was 0.4 mg.

For the expression of *Hydra* Myc2 p15 (106 amino acids; *M*_r_ = 12,646; pI = 10.50), the plasmid pET11d-p15hymyc2 was used. Transformation into bacteria, cultivation, protein induction, and harvesting of bacteria were done as described above for the full-length *Hydra* Myc2 protein. The bacterial pellet was resuspended in 20 ml of buffer E (50 mM NaH_2_PO_4_/Na_2_HPO_4_ pH 7.2, 80 mM NaCl, 1 mM EDTA, 1 mM DTT) and lysed as above. Inclusion bodies were prepared as above using buffer E and then suspended in buffer E supplemented with 7 M urea and separated on a Mono S 5/50 GL cation exchange column (GE Healthcare) in the presence of 7 M urea. Proteins were eluted using a linear gradient from 0 to 0.5 M NaCl at a flow rate of 0.5 ml/ml. Fractions containing the purified recombinant *Hydra* Myc2 p15 protein were pooled and dialyzed against 1 l of buffer E containing 5% (v/v) glycerol for 12 h at 4°C, and concentrated by using an Amicon Ultra centrifugal filter device (MWCO 3,000) (Millipore). The protein was then purified first on a Superdex-75 gel filtration column (GE Healthcare), and then on a Mono S 5/50 GL cation column. *Hydra* Myc2 p15 containing fractions were pooled, concentrated as above and stored in liquid nitrogen. The final yield of purified and soluble *Hydra* Myc2 p15 was 0.1 mg. For mass spectrometry (MS), *Hydra* Myc2 p15 recombinant protein was desalted using Vivaspin 500 PES centrifugal concentrators (MWCO 3,000) (Sartorius, Göttingen, Germany). Concentration (from 500 µl to 100 µl) and addition (400 µl) of 100 mM ammonium acetate in H_2_O (18 MΩ·cm) to the supernatant was repeated 6 times, followed by 6 cycles of concentration and dilution with H_2_O. The final protein concentration of the electrospray ionization (ESI) solution (H_2_O:CH_3_OH 1:1, 1% vol/vol acetic acid) was ∼5 µM. ESI MS using a 7 Tesla Fourier transform ion cyclotron resonance (FT-ICR) instrument (Bruker, Billerica, MA) gave a protein mass value of 12,514.051±0.004 Da (calculated from the most abundant isotopic peaks of (M + nH)^n+^ ions with *n* = 11–21; theoretical mass without initiating methionine: 12,514.055 Da) with internal calibration (error <0.5 ppm) using polyethylene glycol 1,000 as calibrant. The ESI mass spectrum of *Hydra* Myc2 p15 without internal calibrant showed high protein purity (supplementary material Fig. S5A). For sequencing, top-down MS experiments were performed using collisionally activated dissociation (CAD) and electron capture dissociation (ECD) giving *b*, *c*, *y*, and *z*-type fragment ions from cleavage at 103 out of 104 interresidue sites (supplementary material Fig. S5B).

### Protein analysis

The purified recombinant full-length 327-amino acid *Hydra* Myc2 protein was used to generate the rabbit polyclonal antisera α-hy Myc2 according to described procedures (Hartl et al., 2009; Hartl et al., 2010). The antisera α-hy Myc1, α-hy Max, α-Myc-C, and α-Myc-N have been described (Hartl et al., 2010). *In vitro* translation, metabolic labeling of *Hydra* proteins, immunoprecipitation of L-[^35^S]methionine-labeled proteins, SDS/PAGE, and immunoblotting were done as described (Hartl et al., 2009; Hartl et al., 2010). Electrophoretic mobility shift assay (EMSA) analysis and signal quantification were performed as described (Hartl et al., 2010).

### Promoter analysis

Chromatin immunoprecipitation (ChIP) analysis was carried out as described (Reiter et al., 2007; Hartl et al., 2009; Valovka et al., 2013) by using sheared extracts derived from ∼300 *Hydra* animals treated with formaldehyde for 30 min. Immunoprecipitations were performed with specific antibodies followed by PCR amplification of a 281-bp fragment from the *Hydra CAD* regulatory region by using the primer pair 5′-CAGCATAGAGAATGCGGAC-3′/5′-TTTACCTTTCCGGCTGTTTC-3′. Transcriptional transactivation analysis using the luciferase reporter system has been described (Hartl et al., 2009; Valovka et al., 2013). To generate the reporter construct pGL3-hyCAD, a 243-bp segment encompassing nucleotides −97 to +146 of the *Hydra CAD* promoter region was amplified by PCR from genomic DNA using the primer pair 5′-AAATAGTCAATACAATGATTGT-3′/5′-TAGGGAAAATATTTACACACAA-3′, and inserted in the *Kpn*I and *Hind*III sites of the pGL3-Basic vector (Promega, Madison, WI). The expression vector pRc-Myc has been described (Hartl et al., 2009). To create pRc-hymyc2, pRc-hymyc1, and pRc-hymax, the inserts from pA-hymyc2 (see above), or pBS-hymyc1 and pBS-hymax (Hartl et al., 2010) were released with *Xba*I/*Sal*I, *Sal*I or *Xba*I, respectively, and inserted into the eukaryotic pRc/RSV expression vector (Invitrogen, Carlsbad, CA) which had been opened with *Hind*III/*Xba*I.

### Data deposition

The nucleotide sequence of the *Hydra CAD* coding sequence has been deposited in the GenBank database, http://www.ncbi.nlm.nih.gov (accession no. HQ184466).

## RESULTS

### Characterization of *Hydra myc2* and *CAD*

We have recently determined structure and genomic localization of *Hydra myc1* and *myc2* and found that these genes are located on two different loci (Hartl et al., 2010). Interestingly, the *myc2* gene is positioned directly downstream of the putative *CAD* gene (Hartl et al., 2010), encoding a multifunctional enzyme required for pyrimidine synthesis, and representing a well-known transcriptional target of vertebrate Myc proteins (Dang, 1999). Mapping of the *myc2* transcription start site showed that *myc2* mRNA synthesis initiates only 252 nucleotides downstream of the *Hydra CAD* stop codon. The entire coding region of *CAD* was identified by searching the *Hydra* genome database (Chapman et al., 2010) using mammalian CAD protein sequences as templates. Using the sequence information of codons corresponding to the presumed first and last amino acid residues of the *Hydra* CAD protein, the full-length coding region was cloned by using reverse transcriptase-polymerase chain reaction (RT-PCR). The predicted 2213-amino acid *Hydra* CAD protein (accession no. ADN87330) displays the principal topography of vertebrate CAD proteins and shows extensive similarity at the amino acid sequence level.

### Expression of *Hydra myc2* and *CAD* is not restricted to interstitial stem cells

**Fig. 1.**
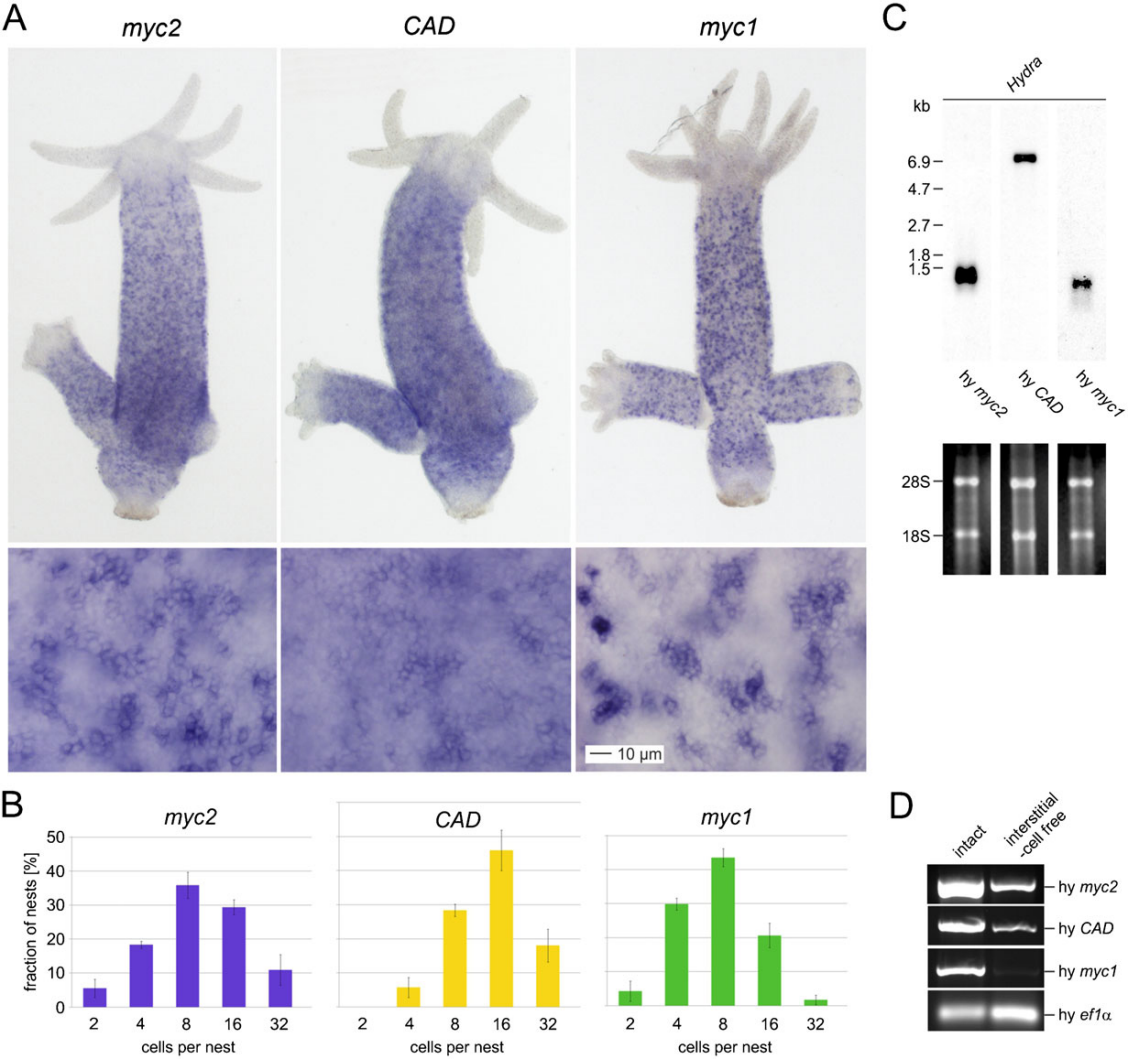
Expression of the *Hydra myc2*, *CAD*, and *myc1* genes. (A) Expression patterns of *myc2*, *CAD*, and *myc1* visualized by *in situ* hybridization. *Hydra myc2*, *CAD*, and *myc1* are expressed in cells throughout the gastric region of intact, budding polyps. Higher magnifications (lower panels) from gastric regions reveal that *myc2*, *CAD*, and *myc1* are up-regulated in interstitial stem cells and proliferating nematoblast nests. In addition, *myc2* and *CAD* are expressed in the surrounding epithelial cells. (B) Quantitative analysis of *myc2*-, *CAD*-, and *myc1*-positive interstitial stem cells and nests of proliferating nematoblasts. The number of cells per nest was determined by visual inspection in the gastric region of in situ hybridized polyps. Each bar represents the mean ± SD from three different counts using various polyps. 100 nests were analyzed in each count. (C) Northern analyses using aliquots (2.0 µg) of poly(A)^+^-selected RNAs from whole *Hydra* animals and *Hydra myc2*, *CAD* or *myc1* specific cDNA probes derived from the coding regions. Ethidium bromide-stained RNAs used for blot analysis are shown below. RNA size markers and positions of residual ribosomal RNAs (28S, 18S) are given on the left site. (D) Detection of *myc2* and *CAD* mRNAs in reverse transcriptase-polymerase chain reaction (RT-PCR) analysis using RNA from interstitial cell-free *Hydra*. Amplification of a specific segment from the *Hydra ef1α* gene served as a control. Scale bar: 10 µm.

To compare the expression patterns of *Hydra myc2* and *CAD* with *Hydra myc1*, *in situ* hybridizations were performed. Similar to *myc1* (Hartl et al., 2010), the *myc2* and *CAD* genes were also strongly transcriptionally activated throughout the gastric region, which represents the area of major cell proliferation in intact polyps (Fig. 1A). *myc2* and *CAD* were not expressed in the differentiated head and foot regions (Fig. 1A). During bud formation, the asexual mode of polyp development, *myc2* and *CAD* started to disappear from the evaginating tip, which continues to form the polyp's head, at stage 4 and from the newly differentiating foot at stage 9 (supplementary material Fig. S1). Observation at higher magnification demonstrated that *myc2* and *CAD* are expressed at a high level in interstitial cells and in nests of proliferating nematocyte precursors (Fig. 1A), as previously shown for *myc1* (Hartl et al., 2010). Quantitative analysis of nest sizes showed a very similar distribution for *myc1* and *myc2* indicating that these two *myc* genes might be expressed in the same interstitial cells (Fig. 1B). Double in situ hybridizations with *myc2* and *myc1* probes confirmed that most of the *myc1*-positive proliferating interstitial cells and nematocyte precursor (nematoblast) nests co-express *myc2* (supplementary material Fig. S2). *CAD* expression was slightly shifted towards larger nest sizes (Fig. 1B) being consistent with its possible role as a potential *Hydra* Myc target gene. Northern analysis using poly(A)^+^-RNAs from whole polyps showed single transcripts of *myc2* (1.4 kb) and *CAD* (7.0 kb), which were more abundantly expressed as compared with the *myc1* transcript (1.2 kb) (Fig. 1C).

In contrast to *Hydra myc1*, whose transcriptional activation is restricted to the interstitial cell lineage, whole mount preparations suggested that *myc2* and *CAD* were also expressed uniformly and at a lower level in epithelial cells of the gastric region (Fig. 1A) To further prove that *myc2* and *CAD* expression is not restricted to the interstitial stem cell system, RT-PCR analysis using RNA from interstitial cell-free animals was performed. Incubation of polyps of the temperature-sensitive mutant strain A10 at elevated temperature leads to specific elimination of the interstitial stem cell system. Whereas under interstitial cell-free conditions no *myc1* expression was observed, *myc2* and *CAD* mRNAs were still abundant (Fig. 1D). Finally, cross sections through the gastric region of in situ hybridized polyps demonstrated that *myc2* and *CAD* are expressed at similar levels in ectodermal and endodermal epithelial cells equivalent to ectodermal and endodermal epithelial expression of *Hydra max* (supplementary material Fig. S3).

### *Hydra myc2* and *CAD* are transcriptionally activated during gametogenesis

**Fig. 2.**
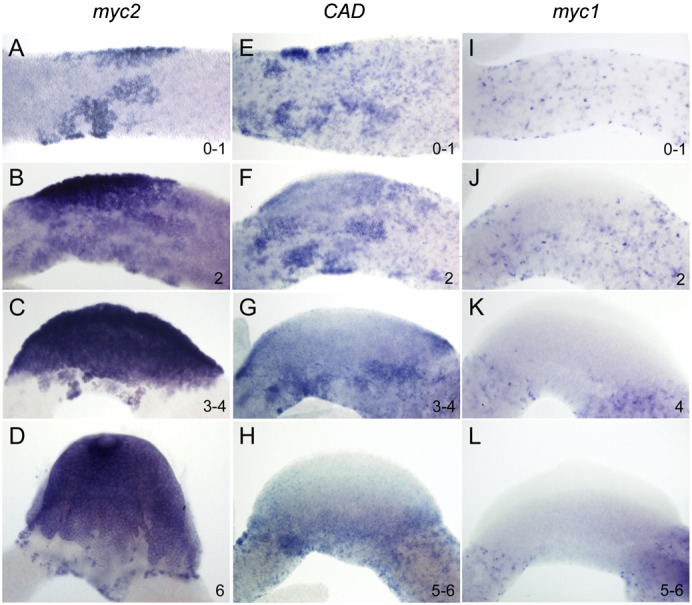
Expression dynamics of *Hydra myc2* (A–D), *CAD* (E–H), and *myc1* (I–L) during oogenesis. *Hydra myc2* is expressed throughout all stages of oogenesis. Transcriptional *myc2* activation is apparent in proliferating and aggregating interstitial cell precursors contributing to formation of the egg field (A,B). Strong *myc2* expression is detectable in the egg patch (C), and in the differentiating oocyte, which characteristically forms pseudopodia to take up nurse cells (D). *CAD* is expressed in proliferating and aggregating interstitial cells in early oogenesis (E). During formation of the egg field, *CAD* expression becomes restricted to the periphery (F,G). In the developing oocyte, *CAD* mRNA is absent but detectable in cells at the periphery (H). *myc1* is not expressed in any cells contributing to oogenesis. Numbers indicate the oogenesis stages (Miller et al., 2000).

In addition to asexual reproduction and steady-state tissue replacement, *Hydra* can undergo sexual reproduction. Eggs and sperm cells develop from a pool of interstitial stem cells located in the gastric region. Interstitial cells undergo several rounds of proliferation forming large patches of egg and sperm precursors in the initial phase of gametogenesis, before differentiation into the mature egg and sperm cells occurs (Littlefield et al., 1985; Munck and David, 1985; Littlefield, 1991). Expression of *myc2*, *CAD* and *myc1* during egg formation was analyzed using sexually induced polyps of the female *Hydra vulgaris* AEP strain. *In situ* hybridizations revealed that *myc2* is strongly expressed throughout all stages of oogenesis (Fig. 2). In stages 0 to 1, *myc2* expression was up-regulated in the large clusters of egg precursor cells, which proliferate and aggregate to form the egg field (Fig. 2A). Egg fields of stages 2 to 4 exhibited an intense *myc2* signal in all regions (Fig. 2B,C), and *myc2* expression levels were also high in the developing stage 6 oocyte (Fig. 2D). At this stage, the oocyte forms finger-like extensions to take up cytoplasm from the surrounding precursors that have been determined to become nurse cells (Honegger et al., 1989; Miller et al., 2000; Alexandrova et al., 2005). The intense staining of the oocyte indicates that *myc2* is used as a maternally deposited factor in the unfertilized egg.

*CAD* is also expressed in clusters of proliferating precursor cells giving rise to the egg field (Fig. 2E). However, further stages of oogenesis show that *CAD* expression is restricted to cells at the periphery of the egg field surrounding the oocyte (Fig. 2F–H). From oogenesis stage 2 on, cells of the egg field stop cycling and start to differentiate into the oocyte or nurse cells (Miller et al., 2000). Since differentiation starts in the center of the egg field and expands towards the periphery, the observed pattern of *CAD* transcription suggests that it is selectively expressed in proliferating oocyte progenitors (Fig. 2F–H). In contrast to *myc2* and *CAD*, there was no expression of *myc1* throughout oogenesis (Fig. 2I–L). Taken together, these findings suggest that *myc2* and *CAD* play distinct roles in female germ cell development, whereas *myc1* does not contribute to egg formation.

**Fig. 3.**
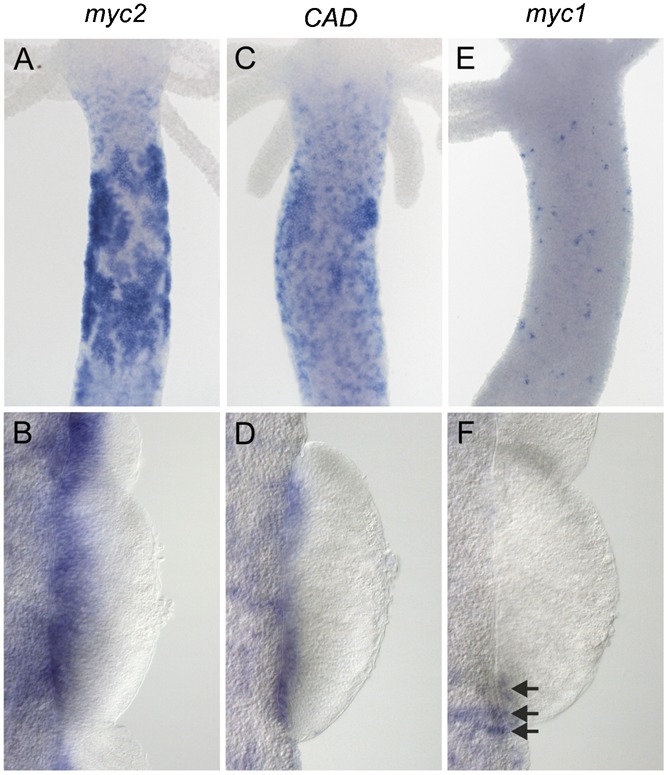
Expression of *Hydra myc2* (A,B), *CAD* (C,D), and *myc1* (E,F) during spermatogenesis. (A,C) Large clusters of early sperm precursor cells strongly expressing *myc2* and *CAD* are present in the upper body column of male polyps. (B,D) *myc2* and *CAD* are expressed in the basal layers of testes representing proliferating spermatocytes. (E,F) *myc1* expression is detectable neither in early stages of spermatogenesis, nor in mature testes. *myc1*-positive somatic interstitial cells in the ectodermal epithelium surrounding the testes are marked by arrowheads in panel F.

Expression analysis of *myc2*, *CAD*, and *myc1* in spermatogenesis was performed in polyps of the male *Hydra vulgaris* PA-2 strain (Fig. 3). Sperm development again starts with intensive proliferation of a pool of interstitial cells in the upper body column resulting in large clusters of sperm cell progenitors (Littlefield et al., 1985; Munck and David, 1985). Thereafter, terminal differentiation of sperm cells takes place in testes formed by ectodermal epithelial cells. Within the testes, differentiating sperm cells are arranged in distinct layers with proliferating spermatogonia and spermatocyte I cells in the proximal third and differentiating spermatocyte II cells, spermatids and mature sperm cells in the more distal parts (Tannreuther, 1909; Brien and Reniers-Decoen, 1950). We found early clusters of interstitial precursor cells showing strong *myc2* and *CAD* expression (Fig. 3A,C). Mature testes showed up-regulation of *myc2* and *CAD* expression in proximal layers directly adjacent to the mesoglea representing the area, where sperm precursor cells undergo their final rounds of proliferation before they differentiate into mature sperm cells (Fig. 3B,D). As during oogenesis, *myc1* was not expressed throughout spermatogenesis. Furthermore, it was clearly absent in mature testes, whereas somatic *myc1*-positive interstitial cells were detectable outside of these male sex organs (Fig. 3E,F). In summary, our expression data support the view that transcriptional activation of *myc2* and *CAD* is associated with proliferation of interstitial and epithelial cells in the *Hydra* polyp during asexual tissue turnover as well as in female and male gamete formation. It was therefore of particular interest to study functional properties of the Myc2 protein in more detail.

### Biochemical properties of the *Hydra* Myc2 protein

To detect the endogenous Myc2 protein encoded by the *myc2* mRNA in *Hydra*, we generated an antiserum specific for the Myc2 protein. For this reason, the coding sequence of *myc2* was inserted into a pET vector, and a recombinant Myc2 protein was expressed in *Escherichia coli* and purified. Polyclonal antibodies generated against the full-length recombinant Myc2 protein were used for immunoprecipitation using lysates from metabolically labelled *Hydra* (supplementary material Fig. S4). For size comparison, *in vitro* translated Myc2 (*M*_r_ 36,961) was included in the analysis. Both *in vitro* translated and endogenous Myc2 proteins display an apparent *M*_r_ of 41,000.

**Fig. 4.**
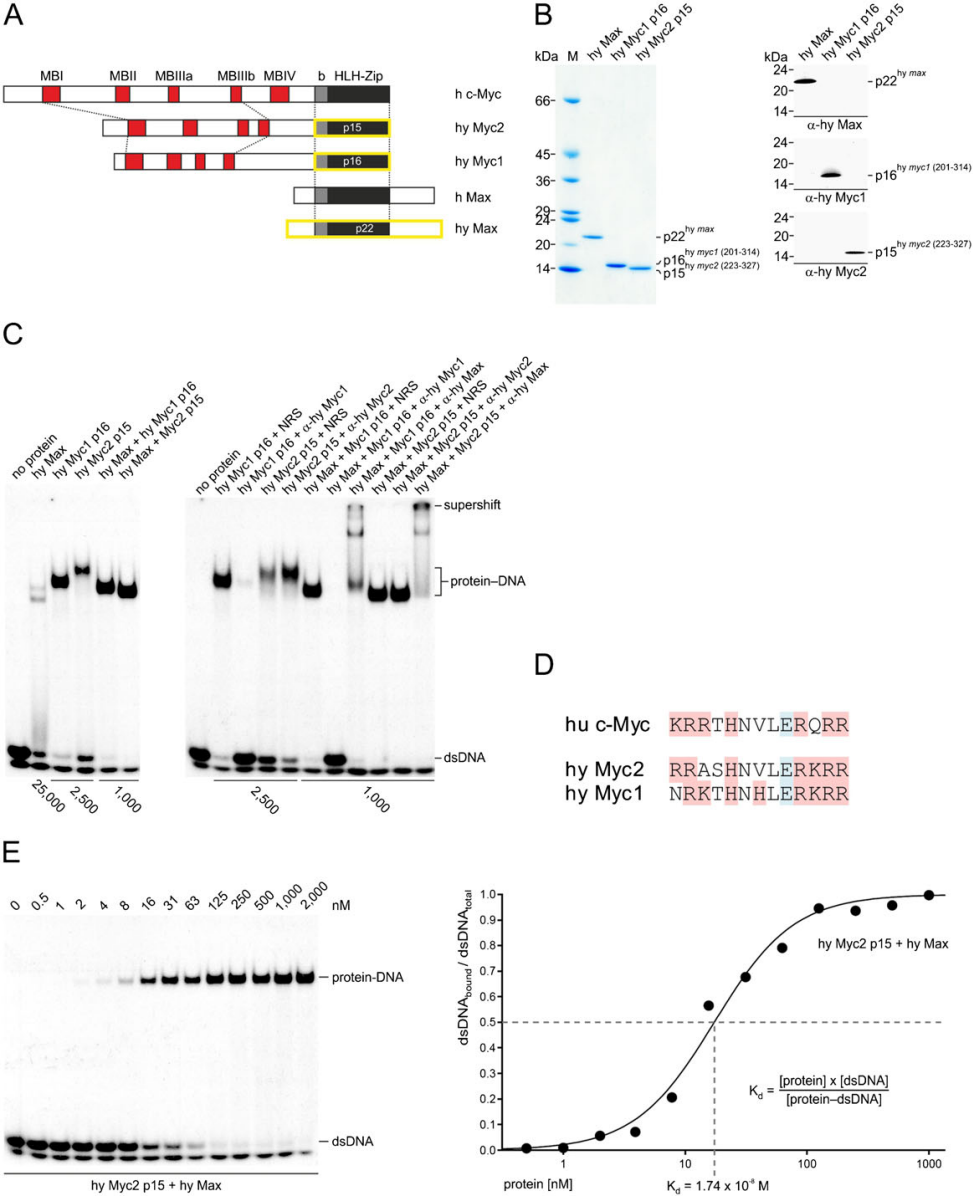
*Hydra* Myc proteins and their biochemical activities. (A) Schematic depiction of the human (h) c-Myc, Max, and the *Hydra* (hy) Myc2, Myc1, and Max protein products (GenBank accession nos.: h c-Myc, NP_002458; h Max, NP_002373; hy Myc2, ADA57607; hy Myc1, ACX32068; hy Max, ACX32069). The positions of conserved basic (b) and helix–loop–helix-zipper (HLH-Zip) domains and of the Myc boxes I–IV (MB) are indicated. The carboxyl-terminal segments of *Hydra* Myc2 (p15) and Myc1 (p16), and the full-length *Hydra* Max (p22) encompassing dimerization and DNA binding domains (b-HLH-Zip) (framed in yellow) were expressed in *Escherichia coli*. (B) SDS-PAGE (5.0–17.5% gradient, wt/vol) of 2-µg (Coomassie brilliant blue staining) or 50-ng (immunoblotting) aliquots of purified recombinant *Hydra* Myc1 p16 (amino acids 201–314), Myc2 p15 (amino acids 223–327), and *Hydra* Max p22. (C) Electrophoretic mobility shift assay (EMSA) using the recombinant proteins shown in panel A and 0.3-ng (25,000 cpm) aliquots of a ^32^P-labeled double-stranded 18-mer oligodeoxynucleotide containing the canonical Myc/Max-binding motif 5′-CACGTG-3′ in the context of an upstream stimulatory factor binding site (E-box USF). Final protein concentrations are indicated below. Left panel: DNA binding of Max, Myc1 p16 and Myc2 p15 homodimers, or Max/Myc1 p16 and Max/Myc2 p15 heterodimers. Right panel: effects of specific antibodies (α) directed against *Hydra* Max, Myc1 p16, or Myc2 p15 to the binding reactions. (D) Amino acid sequence alignment of human (hu) c-Myc and the *Hydra* (hy) Myc2 and Myc1 basic regions. Positively and negatively charged residues are shaded in red or blue, respectively. (E) Determination of the dissociation constant (K_d_) of protein–DNA complexes by EMSA analysis. Titration of a double-stranded oligodeoxynucleotide as in panel C with increasing amounts of *Hydra* Myc2 p15 and *Hydra* Max. Total protein concentrations are indicated at the top (left panel). The ratios of bound DNA to total DNA were determined by phosphorimaging and plotted versus protein concentrations (right panel). Because the experimental conditions led to partial DNA strand separation, only double-stranded DNA was considered for the quantification of unbound DNA. The sigmoidal fit function f(*x*) = 1/{1+exp[(a−*x*)/b]} was used to generate the binding curve. The calculated K_d_ value for the protein–DNA binding reaction is indicated below.

We have shown previously that the principal biochemical functions of Myc already emerged in early metazoans by testing the *Hydra* Myc1 and Max proteins for their potential of specific DNA binding (Hartl et al., 2010). To test Myc2 for DNA binding, the coding sequence of the carboxyl-terminal region of Myc2 containing the dimerization and DNA binding domain (bHLH-Zip) (amino acid residues 223–337) was inserted into a pET vector (Fig. 4A). The proteins Myc2 p15 (*M*_r_ 12,646), Myc1 p16 (*M*_r_ 13,745), and Max p22 (*M*_r_ 20,234) were efficiently expressed, and the soluble fractions purified to homogeneity (Fig. 4B). The identity of recombinant Myc2 p15 was verified by immunoblot analysis, mass spectrometry, and fragment ion mapping (Fig. 4B; supplementary material Fig. S5). This Myc2 p15 protein was then tested together with the purified Myc1 p16 (amino acid residues 201-314) and the purified full-length *Hydra* Max protein (Fig. 4B) (Hartl et al., 2010) using electrophoretic mobility shift analyses (EMSA) (Fig. 4C). The highly conserved basic DNA contact surfaces of Myc2 and Myc1 are very similar in their primary structures (Fig. 4D), and the analysis showed that Myc2 p15 binds to double-stranded DNA containing a consensus Myc binding site almost as efficiently as Myc1 p16 (Fig. 4C), whereas Max p22 displayed lower binding affinity as reported previously (Hartl et al., 2010). The apparent sizes of the Myc2 p15 and Myc1 p16 DNA complexes are higher than expected possibly due to binding of higher order Myc oligomers as proposed previously (Hartl et al., 2010). In agreement with results obtained with Myc1 p16 and Max (Hartl et al., 2010), the DNA affinity is enhanced when an equimolar mixture of Myc2 p15 and Max is used, allowing the formation of heterodimeric protein complexes. To verify the identity of the bound recombinant proteins, the antibodies (α) used for immunoblotting (Fig. 4B) were added to the binding reactions (Fig. 4C). Antibodies can either inhibit or form a ternary protein–DNA complex thereby inducing a supershift. A third possibility is stabilization of a protein–DNA interaction resulting in a shifted band which is more abundant (Carey et al., 2012). As reported previously, α-Myc1 inhibited DNA binding and α-Max induced a supershift (Hartl et al., 2010) proving the identities of the applied recombinant proteins. Addition of the α-Myc2 antibody to the Myc2 binding reaction lead to an increase of bound DNA and induced a slight shift, suggesting stabilization of the Myc2–DNA complex instead of binding inhibition or supershift induction. Presumably, due to binding saturation of the Myc2–Max heterodimer, the stabilizing α-Myc2 effect was not apparent anymore. To quantify the *Hydra* Myc2/Max DNA binding affinity, increasing amounts of proteins were added to constant amounts of DNA in EMSA analyses, the ratios of bound to total DNA were determined, and the dissociation constants (K_d_) for the protein–DNA complex was calculated (Fig. 4E). The K_d_ values for protein–DNA complexes formed by Myc2 p15/Max p22 was determined to 1.74×10^−8^ M which is almost identical to the value previously obtained with the *Hydra* Myc1 p16/Max p22 complex (Hartl et al., 2010).

### *Hydra* Myc interaction with a potential Myc target gene promoter

**Fig. 5.**
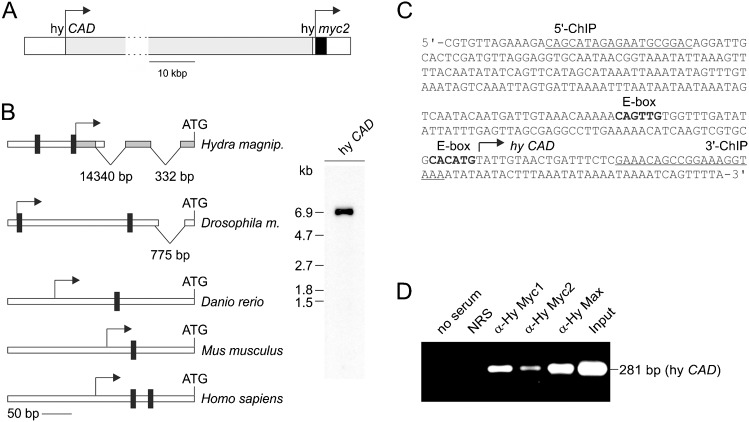
Binding of *Hydra* Myc and Max proteins to the *Hydra CAD* promoter. (A) Topography of the *Hydra magnipapillata* genomic locus (accession nos. NW_002159242, NW_002159544), containing the hy *CAD* and *myc2* genes. The transcription start sites mapped by 5′RACE are indicated by arrows. (B) Left site: schematic structure of the *Hydra magnipapillata CAD* promotor compared with representatives from invertebrate (*drosophila melanogaster*) or vertebrate (*danio rerio*, *mus musculus*, *homo sapiens*) organisms. The positions of consensus Myc binding sites (CANNTG), of the transcription start sites (arrows), and of introns are indicated. Right site: Northern analysis using 2.0 µg of poly(A)^+^-selected RNA from whole *Hydra* animals, and the polymerase chain reaction (PCR) product (shaded in grey) obtained from 5′-rapid amplification of *Hydra CAD* cDNA ends as a probe. The probe specifically detects the 7.0-kb *Hydra CAD* mRNA. (C) Nucleotide sequence of the *Hydra CAD* regulatory region. The transcription start site (arrow), potential binding sites (bold) for the transcription factor Myc (CANNTG), and binding sites for 5′ and 3′ chromatin immunoprecipitation (ChIP) primers (underlined) are indicated. (D) ChIP using chromatin from whole *Hydra* animals. Antisera directed against *Hydra* Myc1, Myc2, and Max were used for precipitation, followed by PCR amplification of a 281-bp fragment from the *CAD* promoter. Reactions with no antiserum, normal rabbit serum (NRS), or total chromatin (Input) were used as controls.

Co-expression of *myc1*, *myc2*, and *CAD* in interstitial stem cells (cf. Fig. 1) suggests the possibility that *Hydra CAD* is regulated by Myc proteins like in vertebrates (Dang, 1999). To test if the *CAD* promoter is bound by Myc1 or Myc2, the *Hydra CAD* transcription start site was first determined by 5′-rapid amplification of cDNA ends (5′RACE) using cDNAs prepared from whole *Hydra* (Fig. 5A). The sequence from the RACE product was aligned with the *Hydra* genome, revealing that there are two introns with sizes of 14,340 bp and 332 bp between the transcription and translation start sites (Fig. 5B). To prove that the 5′-RACE product is derived from the authentic *CAD* mRNA, a Northern analysis was performed using this DNA fragment as a probe (Fig. 5B). Intriguingly, two Myc consensus binding sites (E-boxes) were identified immediately upstream of the transcription start site (Fig. 5C), which is similar to the promoter structures of *CAD* genes in other vertebrate and invertebrate organisms (Fig. 5B). To test if the *CAD* promoter is bound by *Hydra* Myc proteins *in vivo*, chromatin immunoprecipitation (ChIP) analysis was performed using cross-linked chromatin from whole *Hydra* animals followed by PCR amplification of a specific DNA region encompassing the two potential Myc binding sites (Fig. 5D). *Hydra* Myc1, Myc2, and Max proteins were clearly detectable on the *CAD* promoter, whereas no signal was obtained with control reactions using normal rabbit serum or no antibody. This result shows that the putative *Hydra* Myc target *CAD* is bound by Myc/Max complexes. To test if *Hydra* Myc proteins are involved in transcriptional regulation of the *CAD* gene, luciferase reporter assays were performed (supplementary material Fig. S6) using chemically transformed quail cells (QT6) as a cell system. Cells were transiently co-transfected with reporter plasmids containing the *Hydra CAD* promoter inserted into a luciferase reporter vector (pGL3-hyCAD) and expression vectors (pRc) encoding *Hydra* Myc1, Myc2, or Max. To provide the *Hydra* Myc proteins with their authentic dimerization partner, combinations of Myc1, Myc2 and Max expression vectors were also tested (supplementary material Fig. S6A). As positive control, the reporter construct containing the promoter of the v-Myc target *WS5* (pGL3-WS5) (Reiter et al., 2007; Hartl et al., 2009) was included in the analysis (supplementary material Fig. S6B). Ectopic protein expression was verified by immunoblotting. Whereas v-Myc and the *Hydra* Myc1 or Max proteins were efficiently expressed, the Myc2 level was low compared to that of Myc1, although the coding regions of both Myc proteins were inserted into the same vector, and the α-Myc1 and α-Myc2 antisera had a comparable titer (cf. Fig. 4B). No significant transcriptional transactivation of the *CAD* promoter was observed by overexpressing the individual *Hydra* Myc proteins, but co-expression of Myc1 and Max induced moderate transactivation (supplementary material Fig. S6A). In contrast, co-expression of Myc2 and Max did not lead to transactivation possibly due to a lower amount of Myc2 in the transfected cells. Ectopic expression of v-Myc lead to efficient transactivation of the *WS5* promoter as reported previously (supplementary material Fig. S6B) (Reiter et al., 2007; Valovka et al., 2013).

### Transforming potential of *Hydra* Myc2

**Fig. 6.**
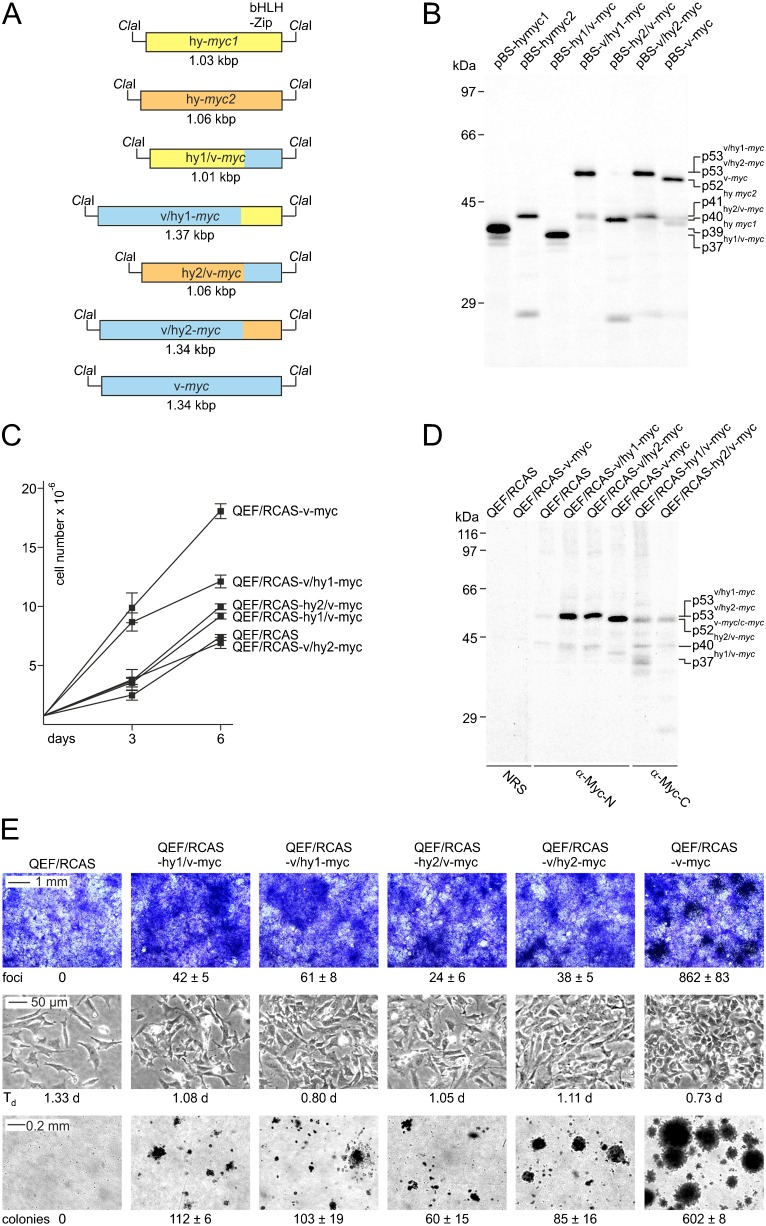
*Hydra* (hy) and viral Myc hybrid proteins display cell transforming activity. (A) Schematic depiction of the v-*myc* (blue), and of hy/v-*myc* and v/hy-*myc* hybrids' coding regions (*Hydra myc1* and *myc2* sequences shown in yellow and orange, respectively). The coding regions were inserted into the unique *Cla*I site of the Bluescript (pBS) vector for *in vitro* transcription/translation, or into the replication-competent retroviral pRCAS vector used for DNA transfection into primary quail embryo fibroblasts (QEF). (B) *In vitro* translation of [^35^S]methionine-labeled proteins encoded by the pBS constructs shown in panel A. (C) Growth of QEFs transfected with the RCAS constructs shown in panel A. After several passages, equal numbers (Y_0_ = 7.5×10^5^) of cells were seeded onto 60-mm dishes and cell numbers (Y) were determined after 3 and 6 days. Standard deviations are indicated by vertical lines. (D) Immunoprecipitation of endogenous c-Myc and ectopic Myc proteins using 1.0×10^7^-cpm aliquots of lysates from [^35^S]methionine-labeled QEFs transfected with the pRCAS constructs shown in panel A, and antibodies directed against amino-terminal [N] or carboxyl-terminal [C] segments of v-Myc, or normal rabbit serum (NRS). Proteins were resolved by SDS-PAGE (10%, wt/vol). (E) Top: QEFs on 60-mm dishes were transfected with 4-µg aliquots of DNA from the pRCAS constructs shown in panel A, kept under agar overlay for 2 weeks, and then stained with eosin methylene blue. Numbers of foci per dish are indicated. Middle: QEFs were transfected with the pRCAS constructs shown in panel A and passaged several times. The doubling times (T_d_) of the cell populations, which are indicated below the phase-contrast micrographs were determined from the average proliferations rates (k) obtained in panel C according to Y = Y_0_(e^k×t^) and T_d_ = ln2/k. Bottom: equal numbers (1.0×10^5^) of transfected and passaged cells were seeded into soft agar and incubated for 2 weeks. Numbers of colonies per 1,000 cells seeded are shown below the bright-field micrographs. Standard deviations are indicated by ± signs. Scale bars: 1 mm (E, top), 50 µm (E, middle), 200 µm (E, bottom).

To explore if similar to *Hydra* Myc1 (Hartl et al., 2010) the *Hydra* Myc2 protein displays some of the principal biological functions of vertebrate Myc, the *myc2* coding region, and hybrids between *myc2* and viral *myc* (v-*myc*) were inserted into the replication-competent retroviral RCAS vector and compared with analogous *myc1*/v-*myc* hybrids for their capacity to induce cell transformation in avian fibroblasts. In these hybrid constructs, the *myc* coding sequences for amino-terminal transcriptional regulation and carboxyl-terminal DNA binding domains had been mutually exchanged (hy2/v-myc, v/hy2-myc, hy1/v-myc, v/hy1-myc). The empty RCAS vector and the RCAS-v-myc construct encoding the 416-amino acid viral Myc (v-Myc) protein were used as controls (Fig. 6A). The identities of the DNA inserts were verified by sequencing and by *in vitro* translation of the protein products (Fig. 6B). *In vitro* translated Myc1 (*M*_r_ 36,024) and Myc2 (*M*_r_ 36,961), and the hybrid proteins hy1/vMyc (*M*_r_ 34,910), v/hy1-Myc (*M*_r_ 47,210), hy2/vMyc (*M*_r_ 36,598), v/hy2-Myc (*M*_r_ 46,432), and the v-Myc protein (*M*_r_ 46,095) display an apparent *M*_r_ of 39,000, 41,000, 37,000, 53,000, 40,000, 53,000, and 52,000, respectively (Fig. 6B). *In vitro* translated Myc2 protein displayed lower chemical stability as compared to Myc1, which could account for the lower ectopic Myc2 levels in transiently transfected cells (cf. supplementary material Fig. S6A). The retroviral constructs were transfected into primary quail embryo fibroblasts (QEF), and cells were passaged several times. Cells transfected with RCAS-v/hy2-myc displayed a lower proliferation rate than the corresponding RCAS-v/hy1-myc cells. Cells transfected with constructs encoding hybrids with amino-terminal *Hydra* Myc portions (hy2/v-myc, hy1/v-myc) still grew slower than RCAS-v/hy1-myc cells but faster than the RCAS control cells (Fig. 6C). Expression of ectopic viral and hybrid Myc proteins and of the endogenous c-Myc protein co-migrating with the v-Myc protein was monitored by immunoprecipitation analysis (Fig. 6D). Ectopic expression of full-length Myc2 did not cause any significant cellular alterations, and Myc1 induced only a marginal increase in cell proliferation as reported previously (Hartl et al., 2010). In contrast, expression of all hybrid proteins led to cell transformation manifested by focus formation, enhanced proliferation, and anchorage-independent growth (Fig. 6E). Hybrid proteins between *Hydra* Myc1 and v-Myc displayed a higher transforming potential compared to analogous hybrids between *Hydra* Myc2 and v-Myc. In particular, the carboxyl-terminus of Myc1 (v/hy Myc1) conferred higher cell proliferation rate, focus and agar colony formation as compared to the corresponding Myc2 hybrid (v/hy Myc2), although protein levels of the ectopic proteins were comparable (Fig. 6D). In agreement with our previous observations (Hartl et al., 2010), transformation by all hybrid proteins was less efficient than that induced by the authentic v-Myc oncoprotein. These results show that both the amino-terminal and the carboxyl-terminal domains of *Hydra* Myc2 are principally capable to substitute for the corresponding regions in the highly oncogenic retroviral v-Myc protein, although the overall transforming potential of Myc2 is lower than that of Myc1.

## DISCUSSION

Although substantial progress has been made over the last 30 years to deeper understand the pleiotropic functions of the Myc protein in cellular proliferation, growth, energy metabolism, differentiation, and apoptosis (Eilers and Eisenman, 2008; Dang, 2012; Conacci-Sorrell et al., 2014), many open questions remain regarding the underlying molecular mechanisms. Furthermore, c-Myc has been identified as one of four gene regulators which are sufficient for reprogramming differentiated adult cells back to a pluripotent stage (Takahashi and Yamanaka, 2006). Because Myc controls multiple transcriptional programs involving the regulation of thousands of genes (Eisenman, 2001; Eilers and Eisenman, 2008; Dang, 2012), the identification of those targets mediating crucial processes in cell proliferation, differentiation, or carcinogenesis has remained difficult.

A possibility to dissect the multiple Myc functions is the usage of genetically established invertebrate model organisms in which *myc* genes have been identified and characterized, like in the freshwater polyp *Hydra*. *Hydra* polyps are among the earliest diverged animal groups used to study the Myc oncoprotein so far. Two bona fide *c-myc* orthologs are present in the *Hydra* genome, *myc1* and *myc2*, encoding proteins with structural conservation in their carboxyl-terminal dimerization and DNA binding regions, and in the amino-terminal transcriptional activation domain (Hartl et al., 2010). Myc1 has been associated with cell cycle progression and ribosome biogenesis in fast proliferating cells of the interstitial stem cell lineage (Hartl et al., 2010). In comparison, expression of *myc2* has not been analyzed so far, and the detailed function of Myc2 has been unresolved. Like *myc1*, *myc2* is expressed in the interstitial stem cell system (cf. Fig. 1D; supplementary material Fig. S3). Double *in situ* hybridization revealed that *myc2* transcriptional activation is detected in a large fraction of *myc1* expressing nests (cf. supplementary material Fig. S2). However, despite this similarity, expression patterns of *myc1* and *myc2* in the interstitial cell lineage are not identical because unique expression of *myc1* was observed particularly in single interstitial stem cells and pairs. Furthermore, our *in situ* hybridization data demonstrate that in addition to proliferating interstitial cells, *myc2* is also transcriptionally activated in slower cycling gamete precursors and proliferating epithelial cells in the gastric region (cf. Figs 1–3). These epithelial cells undergo self-renewal and can be regarded as stem cells (Bode, 1996; Galliot et al., 2006; Bosch et al., 2010; Hobmayer et al., 2012). Notably, these cells also express *max* (Hartl et al., 2010), a gene encoding the natural dimerization partner of Myc proteins. Based on all these findings, we propose that *Hydra* Myc2 might play a basic role in cell proliferation and in maintaining the self-renewal capacity of stem cells. Vertebrate c-Myc is a classic regulator of genes involved in cell cycle progression. We think that this function is conserved in *Hydra* Myc2 based on its biochemical properties and its presence even in proliferating cells that have already obtained a first determination signal, such as cycling nematoblasts and gamete progenitors.

The binding activities of the *Hydra* Myc1 and Myc2 carboxyl-termini are almost indistinguishable (cf. Fig. 4), but differential expression patterns of *myc1* and *myc2* strongly suggest that the functions of these paralogs are not redundant (cf. Figs 1–3). *In situ* hybridization using sexually active animals from female and male strains showed that *myc2* and *CAD* are expressed in proliferating precursors of both egg and sperm cells, which originate from the interstitial stem cell system. Whereas *CAD* expression is restricted to proliferating germ cell progenitors, *myc2* is also up-regulated in differentiated, non-proliferating cells involved in oogenesis including the developing oocyte. Hence the *Hydra* Myc2 protein could represent a candidate maternal factor in the oocyte like in *Xenopus*, where *myc* has been detected as stable maternal mRNA (Godeau et al., 1986; Taylor et al., 1986), and the accumulation of *myc2* mRNA may support cell division in the early *Hydra* embryo.

In mammals, *CAD* represents a bona fide Myc target gene (Dang, 1999) and therefore it was of interest to test if the *CAD* promoter region is also recognized by *Hydra* Myc or Max proteins. Immunoprecipitation analysis using cross-linked chromatin from whole *Hydra* animals showed binding of Myc1, Myc2, and Max to a DNA segment immediately upstream of the *CAD* transcription start site containing two putative Myc binding sites (cf. Fig. 5C). Reporter gene assays showed transactivation by Myc1/Max but not by Myc2/Max complexes (cf. supplementary material Fig. S6A), suggesting that Myc1 possibly activates transcription of *CAD* in interstitial stem cells (cf. Fig. 1). Accordingly, expression of *CAD* in nematoblast nests is temporally delayed when compared with that of *myc1* (cf. Fig. 1A,B). Myc2 apparently does not transactivate the *CAD* gene, but it cannot be excluded that the obtained negative result is due to insufficient protein levels (cf. supplementary material Fig. S6A). Another possibility is that besides Myc other transcription factors are involved in *CAD* gene regulation, as shown previously (Khan et al., 2003), which would explain why we see *CAD* also expressed in distinct *myc1*-negative cells.

Altogether, our data suggest that the *Hydra* Myc2 functions are distinct from Myc1 by possibly promoting the proliferation of various cell types in the interstitial system, and of epithelial stem cells. Presumably, *Hydra Myc2* may have also a distinct function as a master regulator in gametogenesis, suggesting that Myc2 is important for distinct differentiation processes. This may explain why the overall potential of Myc2 to promote cell proliferation is lower than that of Myc1 (cf. Fig. 6). In particular, the oncogenic potential of the carboxyl-terminal dimerization and DNA binding domain of Myc2 is substantially lower than that of Myc1 suggesting that both Myc paralogs regulate different sets of target genes, although the amino acid sequences of the basic regions and their DNA binding affinities are very similar (cf. Fig. 4D). Powerful tools like interfering RNAs or generation of transgenic animals for functional gene analysis in *Hydra* have been established meanwhile (Wittlieb et al., 2006; Chera et al., 2006; Boehm et al., 2012; Ambrosone et al., 2012). Therefore, the metazoan *Hydra* would represent a useful model system to investigate conserved functions of Myc proteins, and of their transcriptional targets in cell proliferation and differentiation.

## Supplementary Material

Supplementary Material
