# Supplementary material for: Hydra myc2, a unique pre-bilaterian member of the myc gene family, is activated in cell proliferation and gametogenesis
Source: Biol Open. 2014 Apr 25;3(5):397–407. doi: 10.1242/bio.20147005 (PMC4021362; doi:10.1242/bio.20147005)
Supplement: Supplementary Material [file supp_3_5_397__index.html]

Hydra myc2, a unique pre-bilaterian member of the myc gene family, is activated in cell proliferation and gametogenesis — Hydra myc2, a unique pre-bilaterian member of the myc gene family, is activated in cell proliferation and gametogenesis — Supplementary Material 

# *Hydra myc2*, a unique pre-bilaterian member of the *myc* gene family, is activated in cell proliferation and gametogenesis

## bio.20147005 Supplementary Material

**Files in this Data Supplement:**

- Supplementary Material - Markus Hartl et al. doi: 10.1242/bio.20147005
