# Supplementary material for: Hydra myc2, a unique pre-bilaterian member of the myc gene family, is activated in cell proliferation and gametogenesis
Source: Biol Open. 2014 Apr 25;3(5):397–407. doi: 10.1242/bio.20147005 (PMC4021362; doi:10.1242/bio.20147005)
Supplement: Supplementary Material [file supp_bio.20147005_bio.20147005-s1.pdf]

Biology Open (2014) 000, 1–11 doi:10.1242/bio.20147005

Supplementary Material
Markus Hartl et al. doi: 10.1242/bio.20147005

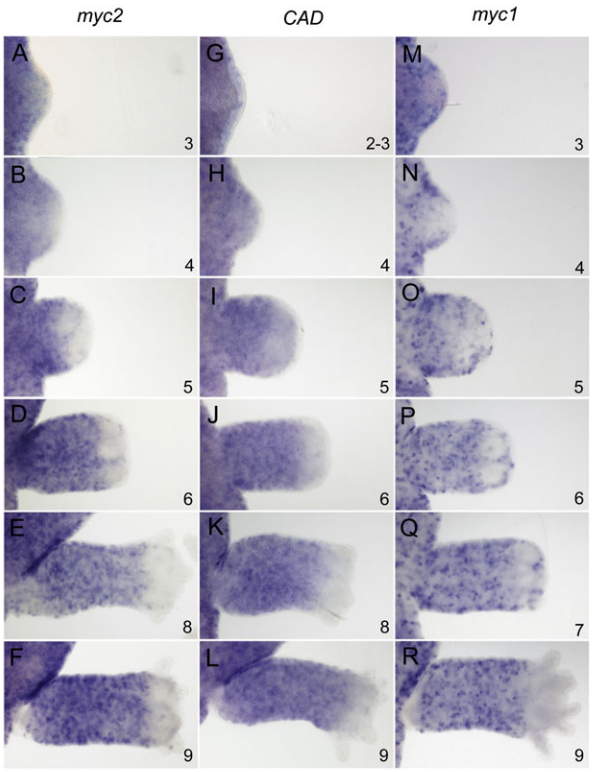

Fig. S1. Expression of *myc2* (A–F), *CAD* (G–L), and *myc1* (M–R) during asexual bud formation using *in situ* hybridizations. For all three genes, expression disappears in regions where terminal differentiation is initiated such as the newly forming hypostome, the evaginating tentacles, and the developing basal disc. Up to bud stage 3, *myc2*-, *CAD*-, and *myc1*-positive cells are located in the distal evaginating tip. Beginning with stage 4, *myc2* and *CAD*-positive cells disappear from the tip and from the positions of newly developing tentacles. Some *myc1*-positive cells remain in the most distal part of the bud up to stage 7. In stage 9, *myc2*-, *CAD*-, and *myc1*-positive cells also disappear from the proximal, basal disc-differentiating part of the bud. Numbers correspond to bud stages.

Biology Open (2014) 000, 1–11 doi:10.1242/bio.20147005

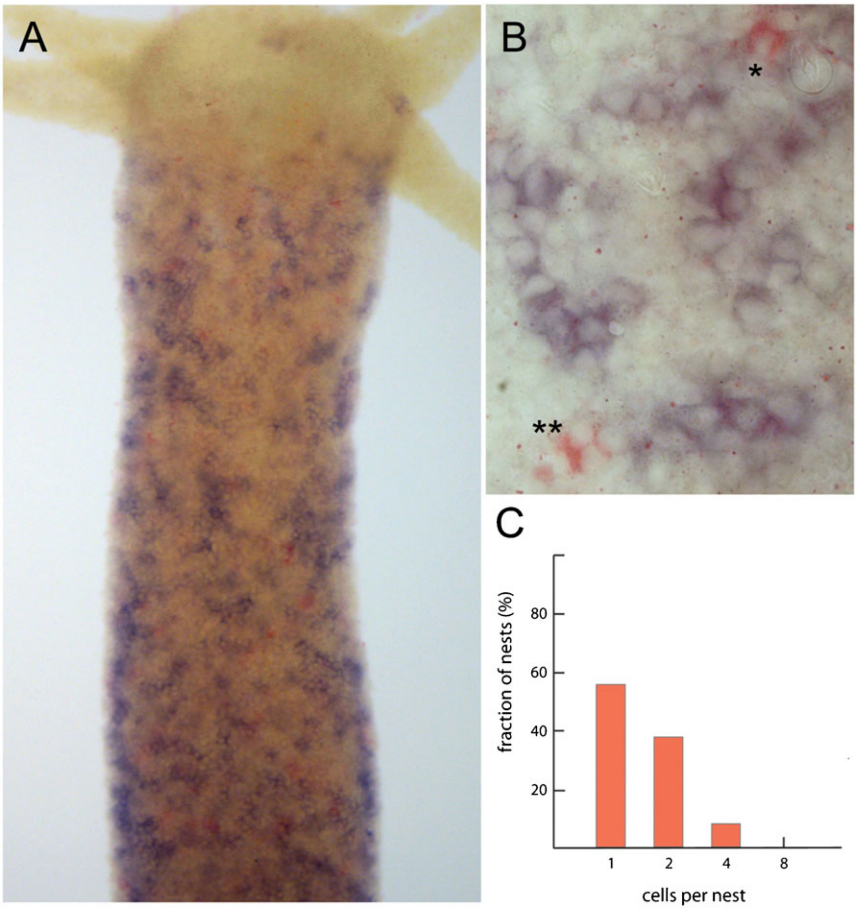

Fig. S2. ***myc1*/*myc2* double *in situ* hybridization.** (A,B) Hybridization with *myc1* (red substrate) and *myc2* (blue substrate) probes resulted in a large amount of cells and nests colored in purple or blue (representing *myc2* + *myc1* or only *myc2* expression, respectively), and a very small number of cells and nests colored in red, in which only *myc1* is expressed. (A) Upper half of a polyp. (B) Magnified view of the gastric region. Notably, the total number of cells and nests stained in red is extremely small in comparison with standard *myc1* in situ polyps as shown in Fig. 1A. Hence, most of the *myc1* expressing cells and nests also express *myc2*. (C) Among cells and nests expressing *myc1* only, quantitative analysis shows primarily single interstitial stem cells (* in panel B) and pairs. Nests containing 4 cells (** in panel B) rarely exclusively express *myc1*, and we did not detect nests with larger cell numbers expressing *myc1* only. The number of cells per nest was determined by visual inspection in the gastric region of in situ hybridized polyps. Bars represent the analysis of 50 red substrate cells or nests from ~15 polyps. Due to the similarity in color between purple substrate (*myc2* + *myc1* expression) and blue substrate (*myc2* expression only), an unambiguous distinction between these two populations was not possible. Therefore, it is not possible with this labeling method to identify cells which express *myc2* only.

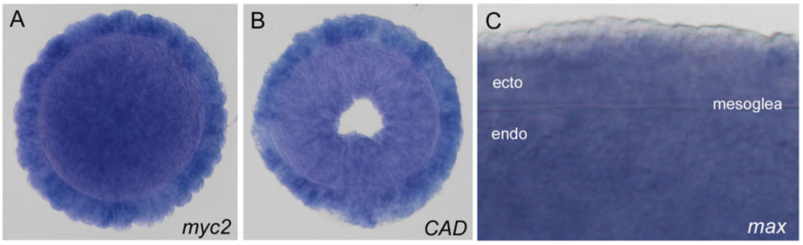

Fig. S3. Expression of *myc2*, *CAD*, and *max* in ectodermal and endodermal epithelial cells. (A,B) About 100-µm thick cross sections of strongly stained in situ hybridized polyps at a mid-gastric position reveal a rather uniform expression level of *myc2* (A) and *CAD* (B) in both epithelial layers. A corresponding cross section through a *myc1* in situ sample lacking expression in the epithelial layers has been published (Ambrosone et al., 2012). (C) Optical cross section of the body wall at midgastric position also shows a rather uniform expression level of *max* in both epithelial layers.

Biology Open (2014) 000, 1–11 doi:10.1242/bio.20147005

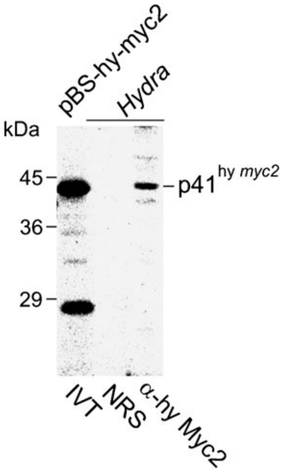

Fig. S4. Expression of the endogenous *Hydra* Myc2 protein. Immunoprecipitation analysis using aliquots (5×10^{6} cpm) of boiled cell extracts from [^{35}S]methionine-labeled *Hydra* animals and a polyclonal antiserum directed against full-length *Hydra* Myc2 recombinant protein (α-hy Myc2), or normal rabbit serum (NRS). For comparison, [^{35}S]methionine-labeled *Hydra* Myc2 p41 protein was also produced by *in vitro* translation (IVT) of a corresponding cDNA cloned in the Bluescript (BS) vector. The 26-kDa polypeptide band in the IVT reaction presumably results from Myc2 degradation. Proteins were analyzed by SDS-PAGE (10%, wt/vol).

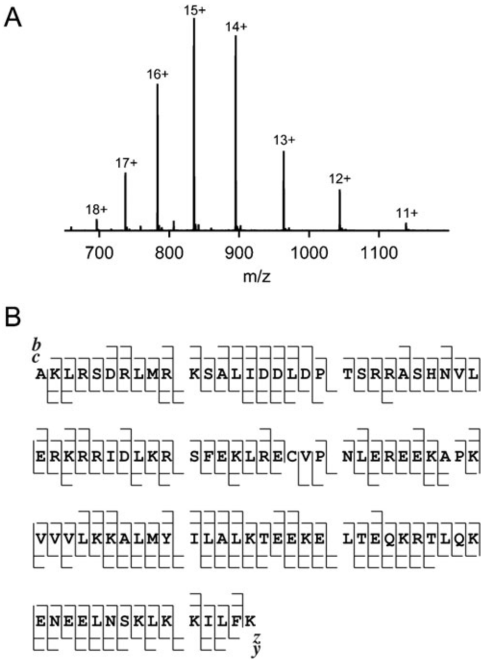

Fig. S5. Mass spectrometry of the *Hydra* Myc2 p15 protein. (A) Electrospray ionization mass spectrum (ESI-MS) of Myc2 p15 recombinant protein with molecular ion charge states indicated; n+ stands for $(M + nH)^{n+}$. (B) Fragment ion map illustrating 99% sequence coverage.

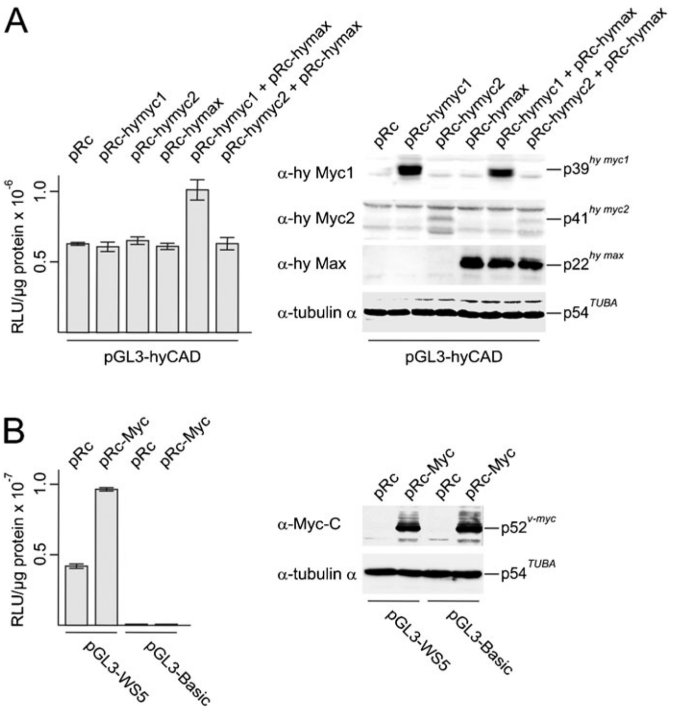

Fig. S6. Transcriptional transactivation of the *Hydra CAD* promoter. (A) Aliquots (3.0 µg) of the pGL3-hyCAD reporter construct were co-transfected with 1.0-µg aliquots of a pRc-derived expression vector encoding the *Hydra* Myc1, Myc2, Max, or the empty expression vector (pRc) into the chemically transformed quail cell line QT6. For triple transfections 0.5 µg of the two expression vectors were transfected together with 3.0 µg of reporter construct. (B) Aliquots (3.0 µg) of the pGL3-WS5 reporter construct containing the promoter of the Myc target gene *WS5* (Hartl et al., 2009), or the empty reporter vector (pGL3-Basic) were co-transfected as above with 1.0-µg aliquots of a pRc-derived expression vector encoding the viral Myc protein (v-Myc). Luciferase activities and protein concentrations were determined for 10-µl aliquots of cell extracts, prepared 24 h after transfection and expressed as relative light units (RLU)/µg protein. Luciferase activities and standard deviations are visualized by bars and vertical lines, respectively. For control of protein expression (right panels), equal amounts of cell extracts (20 µl) were analyzed by SDS-PAGE (10% wt/vol). The ectopic *Hydra* Myc1, Myc2, Max (12% wt/vol gel) and v-Myc proteins, and endogenous tubulin α were detected by immunoblotting.
